# Urinary iodine and thyroid determinants in pregnancy: a follow up study in Sri Lanka

**DOI:** 10.1186/s12884-016-1093-7

**Published:** 2016-10-12

**Authors:** Eric De Zoysa, Manjula Hettiarachchi, Chandrani Liyanage

**Affiliations:** 1 Department of Biochemistry, Faculty of Medicine, University of Ruhuna, Galle, Sri Lanka; 2 Nuclear Medicine Unit, Faculty of Medicine, University of Ruhuna, P.O. Box 70, Galle, Sri Lanka; 3 Department of Community Medicine, Faculty of Medicine, University of Ruhuna, Galle, Sri Lanka

**Keywords:** Urinary iodine, Iodine status in pregnancy, Thyroid stimulating hormone, Thyroid volume, Goitre in pregnancy

## Abstract

**Background:**

Iodine deficiency and thyroid dysfunction during pregnancy is associated with number of adverse outcomes that includes mental and physical disabilities creating a huge human and economic burden in later life. Several indicators are used to assess the iodine status of a population: thyroid size by palpation and/or by ultrasonography, urinary iodine excretion and the blood thyroid hormone profile.

**Methods:**

This prospective study was designed to assess the iodine nutrition during the course of pregnancy with reference to urine iodine concentration (UIC) and thyroid determinants among 425 pregnant women from Galle district, Sri Lanka. UIC was estimated in all three trimesters and thyroid functions were assessed in first and third trimesters.

**Results:**

Median (inter-quartile range IQR) UIC was 170.9 (100.0–261.10) μg/L, 123.80 (73.50–189.50) μg/L and 105.95 (67.00–153.50) μg/L in the first, second and third trimesters respectively (*p* < 0.001). Median thyroid stimulating hormone (TSH) level in the first trimester was 1.30 (0.80–1.80) µIU/mL. This value significantly increased (*p* < 0.001) to 1.60 (1.20–2.10) µIU/mL at the 3^rd^ trimester even though it was maintained within the reference range (0.3 – 5.2 µIU/mL). In the assessment of thyroid gland, 67 (16.0 %) women had palpable or visible goitres and 55 (13.1 %) had a goitre that was palpable but not visible. The median thyroid volume of the sample was 5.16 mL (4.30; 6.10 mL) as measured by ultra sound (US) scanning. In multiple regression analysis after controlling for other independent variables (anthropometric, demographic and biochemical parameters); initial body mass index (BMI), goitre size, thyroid volume and parity had significant correlations with the third trimester urinary iodine levels. The thyroid volume accounted for 4.5 % of the urinary iodine variation.

**Conclusions:**

Even though iodine status was progressively worsening with the advancement of pregnancy and iodized salt consumption has not met with the increasing demand for iodine, it was not reflected in the serum TSH level. Therefore, it is worthwhile to assess the long term effects of rising TSH levels and inadequate iodine nutrition during pregnancy on the offspring to prevent even mild iodine deficiency.

## Background

Maternal iodine deficiency and thyroid dysfunction during pregnancy is associated with number of adverse outcomes. During the first two trimesters of pregnancy, the foetus is entirely dependent on the maternal thyroid hormone supply because the foetal thyroid does not develop until 13–15weeks of gestation [1]. As the foetus progresses into the third trimester, it develops the ability to produce its own thyroid hormones but still dependent on maternal iodine for hormone synthesis [2]. Thyroid hormone is required for normal neuronal migration, myelination, and synaptic transmission and plasticity during fetal and early postnatal life and hypothyroxinemia during these critical periods causes irreversible brain damage with mental retardation and neurologic abnormalities [3]. Further, it was reported that elevated maternal thyroid stimulating hormone (TSH) has been associated with an increased risk of pre-term birth, placental abruption, foetal death, and impaired neurological development in the child [4].

Urine iodine concentration (UIC) remains the gold standard in monitoring iodine nutrition at the population level [5]. Serum TSH can be used as an indicator of iodine nutrition, because it is determined mainly by the level of circulating thyroid hormone, which in turn reflects iodine intake. However, in older children and adults, although serum TSH may be slightly increased due to iodine deficiency, values often remain within the normal range [3]. As a single hormone determinant, serum TSH provides the most sensitive index to reliably detect thyroid function abnormalities [6]. Therefore, this study was designed to assess the iodine nutrition of pregnant women prospectively during the course of the pregnancy with reference to the urine iodine concentration and the thyroid functions. It was also aimed at evaluating the effectiveness of salt iodization programme in maintaining iodine nutrition among pregnant women in Sri Lanka.

## Methods

This prospective cohort study was conducted in the Galle district in the South of Sri Lanka during the period from July 2012 to September 2014 with approval of the Ethics Review Committee of the Faculty of Medicine, University of Ruhuna, Sri Lanka. The calculated sample size was 385. A possible prevalence of 50 % iodine deficiency among pregnant women was assumed and it was further inflated by 10 % to cover up possible termination of pregnancies and drop outs during the study period. Altogether 425 pregnant women were enrolled in the study. With an informed consent, pregnant women with gestational age: ≤ 12 weeks (calculated by the date of last menstrual period) were included in the study. Pregnant women with previous history of thyroid and renal diseases were excluded. Data collection was done by using a pretested interviewer administered questionnaire.

An examination of the neck was done to assess the size of the thyroid gland by palpation and by ultrasound (US) scanning at their antenatal clinics using a portable scanner with a high frequency linier 7.5–10 MHz transducer. The volume of the thyroid gland was calculated from the measurements of the depth (d), the width (w), and the length (l) of each lobe by the formula recommended by World Health Organization [7]. The thyroid volume taken was the sum of volumes of both lobes. The volume of the isthmus was not included as the measurement did not include the thyroid capsule (hyper echoic to the gland tissue) or the thyroid isthmus.

Clean mid stream spot urine samples were collected from study subjects at the study entry, at the second (at or around 24^th^ weeks of gestation) and at the third (at or around 36^th^ weeks of gestation) trimesters to iodine free, screw capped plastic containers. Collected urine samples were transported in cold packs to the laboratory and stored at −80 °C until the analysis was completed. Urine samples were analyzed in duplicate within a week of collection using ammonium persulfate digestion method [7] at the iodine contamination free laboratory. Blood samples of 5 mL were drawn from each participant, at the study entry and at the third trimester and serum was separated and stored at -80 °C until analysis for thyroid profile (TSH, free thyroxine and thyroglobulin) was done. Thyroid profile was measured using enzyme-linked immunosorbent assay (ELISA) kits provided by MP Biochemicals, USA. The reference values recommended by the American Thyroid association (ATA) for TSH during pregnancy, 0.1–2.5 µIU/mL in the first trimester and 0.3–3.0 µIU/mL in the third trimester [8] were used for comparison.

## Statistical methods

Results of laboratory analysis are presented as mean ± standard deviation (SD) or median with inter-quartile range (IQR). For variables exhibiting a skewed distribution, the median was used as the measure of central tendency together with the IQR. Wilcoxon signed ranks test was used to compare biochemical parameters at different intervals and linear regression analysis to test for correlations between UIC with other variables. A *p-*value less than 0.05 was considered as significant. Statistical analyses were performed using the SPSS version 16 (SPSS, Chicago, IL).

## Results

The mean age of the 425 pregnant women included in the study was 28.2 years (range 16 – 44 years). However, 52 pregnant women were lost on follow up,22 had early termination of pregnancy; 10 did not attend clinics; 14 moved from the region; 6 refused to attend phlebotomy sessions). Therefore, only 389 and 373 urine samples were analysed in the second trimester and the third trimester respectively. Mean ± SD height and weight of the study subjects were 154.2 ± 6.1 cm and 51.7 ± 10.1Kg respectively. The mean BMI of the women at study entry was 22.2 kg/m^2^ and 32.4 % of the women were classified as undernourished (BMI <18.5 kg/m^2^). Further, 166 (39.1 %) women were in their first pregnancy. 301 (70.8 %) were Sinhalese and the rest (*n* = 124, 29.2 %) were Muslims. Even though 322 (75.76 %) women had completed education up to secondary level, only 104 (24.5 %) were employed and the demographic data of the study sampleis given in Table 1.

**Table 1:** Baseline characteristics of study participants^a^

Characteristics	*n*	%
Age in years		
16 – 25	138	32.6
26 – 35	242	57.0
≥ 36	45	10.4
Parity		
1	166	39.1
2–3	235	55.3
≥ 4	24	5.6
Level of education		
Primary & Secondary	100	23.7
Passed O/L	148	34.3
Passed A/L & above	177	42.0
Social class		
Class – I & II	68	16.0
Class – III & IV	213	50.2
Class – V & VI	144	33.8
Thyroid size		
Grade 0	354	84.1
Grade 1	55	13.1
Grade 2	12	2.8

^**a**^there were 425 participants at the study entry

In the assessment of thyroid gland, it was revealed that 67 (16.0 %) women had palpable or visible goitres and 55 (13.1 %) of them had a goitre that was palpable but not visible. The median thyroid volume of the sample was 5.16 mL (IQR 4.30; 6.10 mL) as measured by US scanning. The thyroid volume has shown a significant direct relationship with the gland size since the mean ± SD volume among grade 0 was 5.25 ± 1.5 ml; grade 1 was 5.89 ± 1.6 ml whereas grade 2 was 8.11 ± 6.1 ml (*t* test 16.9, *p* < 0.001). Further, it was evident that mean thyroid volume (5.10 ± 1.9 ml) of women who were in their first pregnancy was significantly low (*t* test 14.1, *p* < 0.001) when compared with that of women who were in their second or subsequent pregnancies (mean volume of 5.61 ± 1.9 ml). Further, the younger women (age <25 years) had significantly lower mean thyroid volume (*t* test 5.08, *p* = 0.04) when compared with older group (age > 25.1 years) of pregnant women (5.11 ± 1.4 ml vs. 5.61 ± 2.3 ml respectively).

The median UIC of women dropped significantly with progression of pregnancy (*p* < 0.001), and the concentrations varied widely at each trimester (Table 2). The median UIC of the women in first trimester was 170.9 μg/L (IQR 100.00 – 261.10 μg/L). It was dropped to 123.80 μg/L (IQR 73.50 – 189.50 μg/L) at the second trimester (Z = 7.28; *p* < 0.001). A further drop of UIC at the end of 3^rd^ trimester to 105.00 μg/L (IQR 67.00 – 153.50 μg/L) was evident with a statistically significant reduction (Z = 4.11; *p* < 0.001). The first trimester urinary iodine level has a significant positive correlation (r = 0.124, *p* = 0.02) with women’s weight, but not with any other parameters (demographic & biochemical) at the study entry.

**Table 2:** Biochemical parameters during pregnancy^a^

	First trimester	Second trimester	Third trimester
*n*	425	389	373
Weeks (weeks)	9.5 ± 1.8	25.3 ± 1.4	36.6 ± 0.6
Urinary iodine (μg/L)			
Median	170.90	123.80	105.00
25^th^ – 75^th^ percentiles	100.00 – 261.10	73.50 – 189.50	67.00 – 153.50
10^th^ – 90^th^ percentiles	68.40 – 329.70	46.00 – 250.30	49.20 – 218.80
Total range	6.30 – 491.70	5.80 – 466.70	16.20 – 505.50
n (%) <50 μg/L^b^	18 (4.3)	36 (9.6)	40 (10.8)
n (%) <150 μg/L^b^	159 (37.4)	168 (45.0)	232 (62.2)
n (%) >250 μg/L^b^	124 (29.1)	37 (10.7)	26 (6.9)
Serum TSH (µIU/mL)			
Median	1.30		1.60
25^th^ – 75^th^ percentiles	0.80 – 1.90		1.20 – 2.10
10^th^ – 90^th^ percentiles	0.50 – 2.50		0.80 – 2.98
Total range	0.40 – 16.60		0.50 – 11.60
Serum fT_4_ (pmol/L)			
Median	18.00		15.45
25^th^ – 75^th^ percentiles	15.45 – 23.20		11.60 – 21.80
10^th^ – 90^th^ percentiles	10.60 – 28.30		10.30 – 28.30
Total range	10.30 – 42.50		10.30 – 39.90
Serum Thyroglobulin (μg/L)			
Median	5.09		
25^th^ – 75^th^ percentiles	1.97 – 7.37		
10^th^ – 90^th^ percentiles	0.38 – 15.65		
Total range	0.14 – 42.0		

^a^Urinary iodine levels was assessed at each trimester, except for serum thyroglobulin other biochemical parameters were assessed at the study entry (first trimester) and towards the end of third trimester

^b^World Health Organization’s criteria for assessing iodine status on population basis: for pregnant women moderate iodine deficiency <50 μg/L insufficient intake <150 μg/L and above requirement >250 μg/L

The serum TSH level of the subjects in their 1^st^ trimester was 1.3 µIU/mL (IQR 0.8–1.8 µIU/mL). At the end of the 3^rd^ trimester it was significantly increased to 1.6 µIU/mL (IQR 1.2–2.1 µIU/mL) (Z-test = 6.4, *p* < 0.001). When method specific reference values for pregnancy (0.3–5.2 µIU/mL) is used to determine the prevalence of hypothyroidism, 10 (2.4 %) and 2 (0.5 %) pregnant women with serum TSH >5.2 µIU/mL at the first trimester and third trimester respectively were identified. None of them had serum TSH values below the lower limit of the reference range. As per ATA reference values, 47 (11.2 %) subjects in the first trimester and 32 (8.7 %) at the third trimester were hypothyroid, indicating a higher prevalence. Median vales of fT_4_ for first and third trimesters were 18.0 pmol/L and 15.5 pmol/L (*p* = 0.002) respectively. None of the subjects were found with fT_4_ values below the lower limit (<6.4 pmol/L) of the method specific reference (hypothyroid) range in both first and third trimesters. Serum thyroglobulin level was also determined to assess the maternal iodine nutrition among pregnant women at the study entry and the median thyroglobulin level was 5.09 μg/L. Of the sample, 47 subjects had thyroglobulin level < 3.0 μg/L.

We performed multiple regression analysis with stepwise elimination (forward and backward) to assess the effect of selected explanatory variables on the urinary iodine levels at first and third trimester respectively (Table 3). After controlling for other independent variables (anthropometry, demographic and biochemical parameters), only weight was found to have statistically significant correlations with the first trimester urinary iodine level. However, initial body mass index (BMI), goitre size, thyroid volume and parity had significant correlations with the third trimester urinary iodine levels. Furthermore, thyroid volume accounted for 4.5 % of the urinary iodine variation (*r* ^2^ = 0.045).

**Table 3:** Multiple regression equation with urinary iodine levels at each trimester as the dependent variables and selected independent variables among study subjects^a^

Variable	Urinary iodine (T_1_)		Urinary iodine (T_3_)	
	ß	*p*-value	ß	*p*-value
Anthropometry				
Weight	0.157	0.006	0.090	0.37
Height	−0.033	0.56	0.032	0.60
BMI	0.005	0.96	0.167	0.005
Thyroid volume	−0.031	0.60	0.117	0.03
Thyroid size	−0.081	0.15	0.136	0.02
Demography				
Social class	0.074	0.20	−0.010	0.87
Education level	0.019	0.73	−0.008	0.90
Parity	0.062	0.28	−0.123	0.04
Age in years	−0.002	0.97	−0.009	0.89
Period of gestation	−0.082	0.15	0.044	0.45
Biochemical analysis				
Serum TSH^b^	−0.066	0.25	−0.006	0.92
Serum fT_4_^b^	0.040	0.49	−0.061	0.29
Serum thyroglobulin^c^	−0.045	0.62	−0.069	0.51
*p* (model)	0.006		<0.001	
*r* ^2^	0.025		0.045	

^a^Anthropometry and demographic data were collected at study entry (first trimester) were compared with urinary iodine level of first and third trimester

^b^These biochemical parameters were assessed separately first trimester data with first trimester urinary iodine and third trimester data with third trimester urinary iodine respectively

^c^Serum thyroglobulin level was measured at study entry and compared with urinary iodine level of first and third trimester

## Discussion

The present study shows that the iodine level of pregnant women in Galle, Sri Lanka was adequate at the onset of pregnancy where as there were only 4.3 % pregnant women with moderate iodine deficiency (urinary iodine <50.0 μg/L). However, with the progression of pregnancy there was steady decline in maternal urinary iodine status and increase in deficiency status and the observed difference was highly significant (*p* < 0.001). Such progressive decline in urine iodine level with the advancement of gestational age was quite compatible with the results of studies done in Bangladesh [9], Congo [10], Nigeria [11] and in some other countries too [12–14]. This reduction in UI concentration can be attributed to the miss-match between the increased demand and the supply of iodine with the advancement of the pregnancy [15]. A recent study done in France showed that median urine iodine level in the first trimester of the pregnancy was low (116.0 μg/L) [4] and similar finding was reported from Thailand (median UI level of 108.0 μg/L in 1^st^ trimester pregnant women) too [16]. It was evident that even in iodine-sufficient countries pregnant women may be at risk of mild deficiency; because of the major physiological changes in thyroid function occurring during pregnancy leading to increased loss of iodine and the need of much higher iodine supply [17].

In contrast, results of national health and nutrition examination survey in USA showed that UI levels were increased in the second and third trimesters when compared to the first trimester [18]. This increase in iodine status was attributed to the usage of supplements containing iodine during pregnancy. However, Sri Lanka did not prescribe supplements containing iodine during pregnancy as a part of their antenatal management. Further, a recent study done in India showed that mean UI levels above normal in all trimesters, with a peak in mid-pregnancy with no inter-trimester variation and only a very few (2 %) had iodine deficiency [19].

An assessment of thyroid functions concurrently with urinary iodine level would give a clear picture on maternal iodine nutrition. Thyroid dysfunction during pregnancy was classified from subclinical hypothyroidism (SCH) to overt hypothyroidism (OH). The SCH was defined as having serum TSH level in the range of 2.5–10.0 µIU/mL with a normal fT_4_ concentration. The distinction of OH from SCH was important because around 2.0–2.5 % of healthy non pregnant women in United States were considered as having SCH, but it would be anticipated that such percentages would be higher in areas of iodine insufficiency [8]. Since the SCH is a biochemical diagnosis, the symptoms may be mild, non-specific and may mimic typical symptoms in pregnancy. The increase in serum TSH level with the advancement of pregnancy in our study was compatible with the findings of the study done in Bangladesh [9]. A recent study done on pregnant women (in their second trimester) in Iran showed that the prevalence of hypothyroidism in pregnant woman was 13.7 % and the prevalence of hyperthyroidism was 1.5 % [20]. Further, Yassaee et al., [21] in 2014 reported 4.15 % prevalence of SCH in the same country. The prevalence of hypothyroidism among north Indian pregnant women was14.3 % during the first trimester [22].

Sri Lanka was categorized as a country with satisfactory iodine nutrition status and the coverage of adequate iodized salt usage was >90 % [23]. Further, Sri Lanka was considered as having achieved all the criteria of WHO/ICCIDD for monitoring progress towards sustainable elimination of IDD. However, no national level studies have been conducted to assess the iodine nutrition in pregnant women in Sri Lanka up to 2010, when a study revealed that the overall median urine iodine concentration among pregnant women was 113.7 μg/L indicating an iodine deficiency in Sri Lankan pregnant women [24]. This cross sectional study showed that pregnant women who were in the second and third trimesters had lower UI level than first trimester pregnant women. To our knowledge, present study is the first prospective study done in Sri Lanka to assess the gestational changes in iodine nutrition along with the thyroid profile assessment.

In this group of pregnant women, UI level was significantly reduced over the period of gestation and almost three fourth of the pregnant women were iodine deficient (<150.0 μg/L) towards the end of the pregnancy. However, this iodine deficiency status was apparently not reflected by the serum TSH level. Even though iodine deficiency was a significant problem in this sample, prevalence of severe iodine deficiency (<50.0 μg/L) was very low. Results of the present study confirmed that pregnant women in this study population were having mild iodine deficiency with normal TSH level. Maintaining median serum TSH level within the reference range (increased serum TSH values remained within the normal range) with progressive increase in iodine deficiency in this study sample may be explained by this mild iodine deficiency status and the foetuses of mothers who are apparently euthyroid may have insufficient T_4_ for its normal neurodevelopment [25]. These conditions have been found to be related to mild and subclinical cognitive and psychomotor deficits in neonates, infants and children [17] and it highlights the importance of maintaining proper iodine nutrition throughout the pregnancy to ensure optimal maternal thyroid function for a better pregnancy outcome.

This study has some important points. The design was suited to evaluate changes in iodine nutrition during the course of the pregnancy because we measured the urine iodine excretion in each trimester in the same study subject whereas many of the other studies were done in cross- sectional manner [18, 24, 26, 27]. Pregnant women of the study sample were not given iodine supplements and it mainly explores the effectiveness of iodized salt, diet and water as sources of iodine during pregnancy. The main limitations were the lack of information concerning iodine concentrations in edible salts utilized at each household and recruitment of pregnant women from a health division close to the main city of the Southern province where there may be a difference in level of education, nutritional status and social class compared to the general population of the country. Therefore the results may not be generalized to the Sri Lankan pregnant population. It would have been useful to have a non-pregnant control group from the same area to ascertain whether lower UI concentrations during pregnancy could be attributed to pregnancy itself or to different dietary choices among them.

## Conclusions

In conclusion, pregnant women in Galle, Sri Lanka have generally inadequate urinary iodine excretion during progression of the pregnancy. They should be encouraged to either consume iodine-rich food or to take appropriate multiple micronutrient capsules including iodine to improve their iodine status during pregnancy. However, existing iodine supplement through salt iodization programme should be continued and monitored strictly with a close surveillance to maintain a satisfactory iodine status in the pregnant women. Further studies are needed to clarify the potential impact of the women’s consistently low or excessive iodine intake and benefits or adverse effects of iodine supplementation on the health of their developing offspring.

## Abbreviations

ATA: American thyroid association
BMI: Body mass index
ELISA: Enzyme-linked immunosorbent assay
IDD: Iodine deficiency disorders
IQR: Interquartile range
OH: Overt hypothyroidism
SCH: Subclinical hypothyroidism
SD: Standard deviation
TSH: Thyroid stimulating hormone
UI: Urinary iodine
UIC: Urine iodine concentration
US: Ultra sound
